# Appendiceal Mucocele Presenting as a Cecal Bulge on Surveillance Colonoscopy

**DOI:** 10.7759/cureus.69283

**Published:** 2024-09-12

**Authors:** Monique E Le Donne, Michael Herman

**Affiliations:** 1 Surgery, Lake Erie College of Osteopathic Medicine, Bradenton, USA; 2 Gastroenterology, Borland Groover, Jacksonville, USA

**Keywords:** appendiceal mucocele, appendix filled with mucin, appendix tumors, colonoscopy surveillance, mucinous neoplasms, mucocele of the appendix, screening colonoscopy

## Abstract

Appendiceal mucoceles are rare tumors with diverse presentations and clinical implications. Generally, mucoceles are discovered on imaging or intraoperatively, but, rarely, can be found on colonoscopy. Appendectomy is the recommended next step in management, followed by subsequent treatment according to guidelines dictated by pathology findings. We present the case of a 52-year-old female undergoing routine colonoscopy screening who had incidental findings of a cecal bulge at the appendiceal orifice. The patient was entirely asymptomatic with an unremarkable medical history. Further workup suggested appendiceal mucocele and she was referred to surgery. After an appendectomy, pathology confirmed simple appendiceal mucocele. The patient made a full recovery and continued routine colonoscopy screening. This case demonstrates the variance in the clinical presentation of appendiceal mucocele and the possibility of discovery on routine screening colonoscopy.

## Introduction

Several types of tumors occur in the appendix, including adenomas, mucin-producing or non-mucin-producing adenocarcinomas, and carcinoid tumors. Mucin-producing tumors, otherwise known as mucoceles, most often occur in the sixth decade of life and in females [1,2]. New research suggests appendiceal mucocele pathogenesis sometimes involves mutations in *KRAS* and *GNAS*, much like papillary mucinous neoplasms of the pancreas [3]. Appendiceal mucoceles may present similar to appendicitis clinically or have no symptoms at all. Asymptomatic mucoceles are generally an incidental finding [1]. Mucoceles are rare, accounting for just 0.3-0.7% of all appendix pathologies, and 3,500 cases per year in the United States [4]. Considering their potentially fatal sequelae when high grade, such as pseudomyxoma peritonei, it is important to recognize mucoceles when found on routine screening colonoscopy [1]. This report presents a case of an asymptomatic appendiceal mucocele found on routine surveillance colonoscopy appearing as a cecal bulge.

## Case presentation

**Figure 1:**
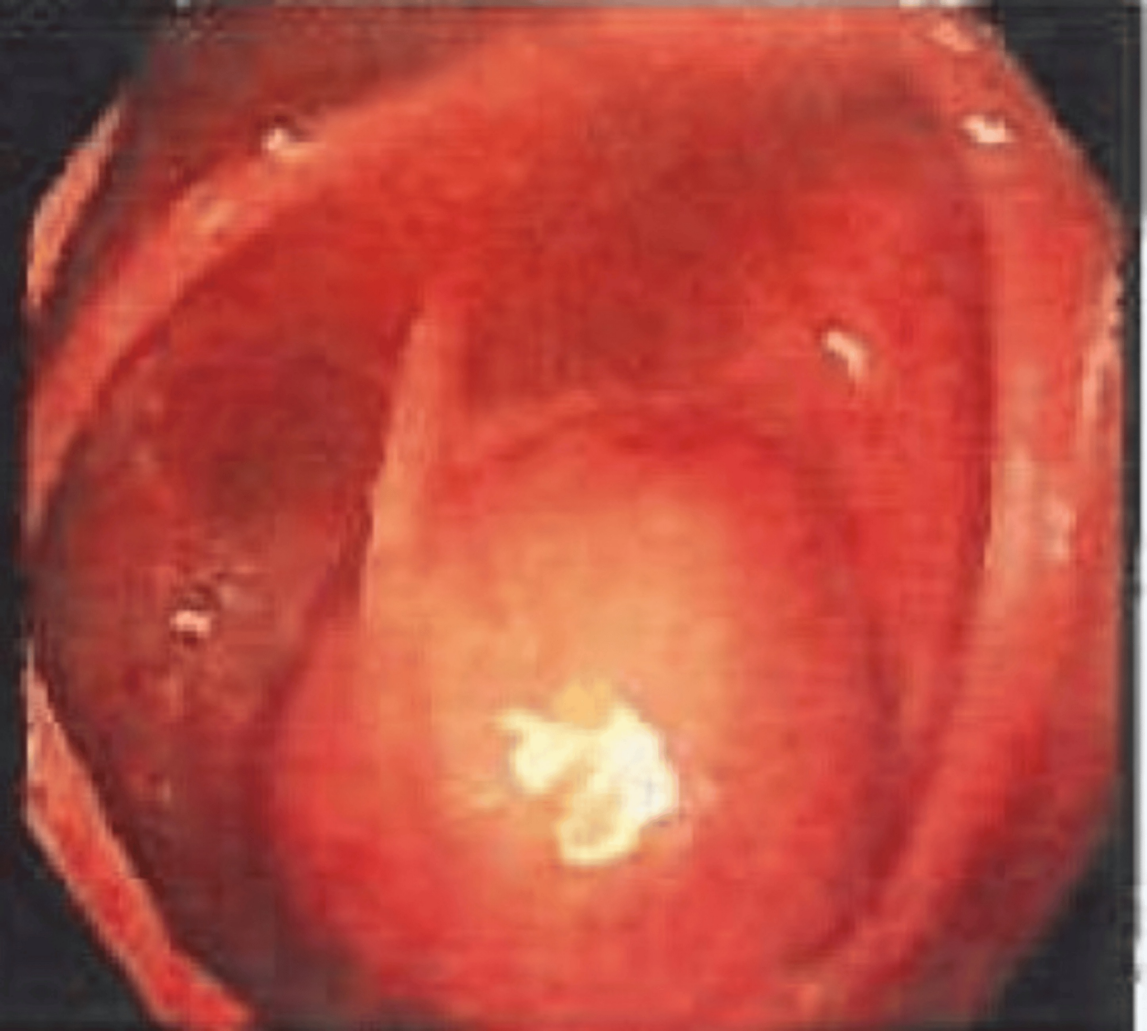
Cecal bulge at the appendiceal orifice seen on routine colonoscopy.

**Figure 2:**
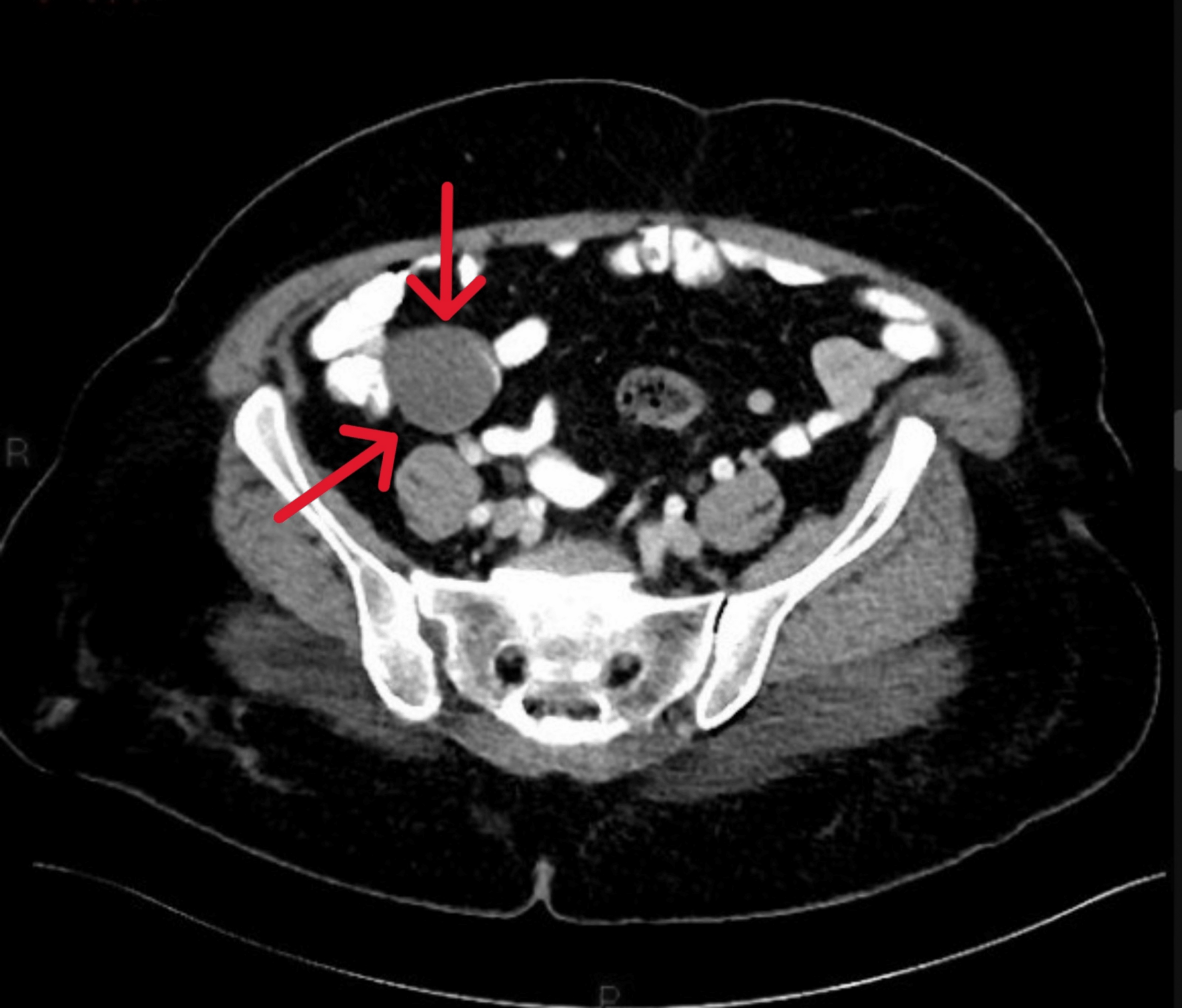
CT of the abdomen with contrast demonstrating hypodense appendiceal mucocele in transverse view.

**Figure 3:**
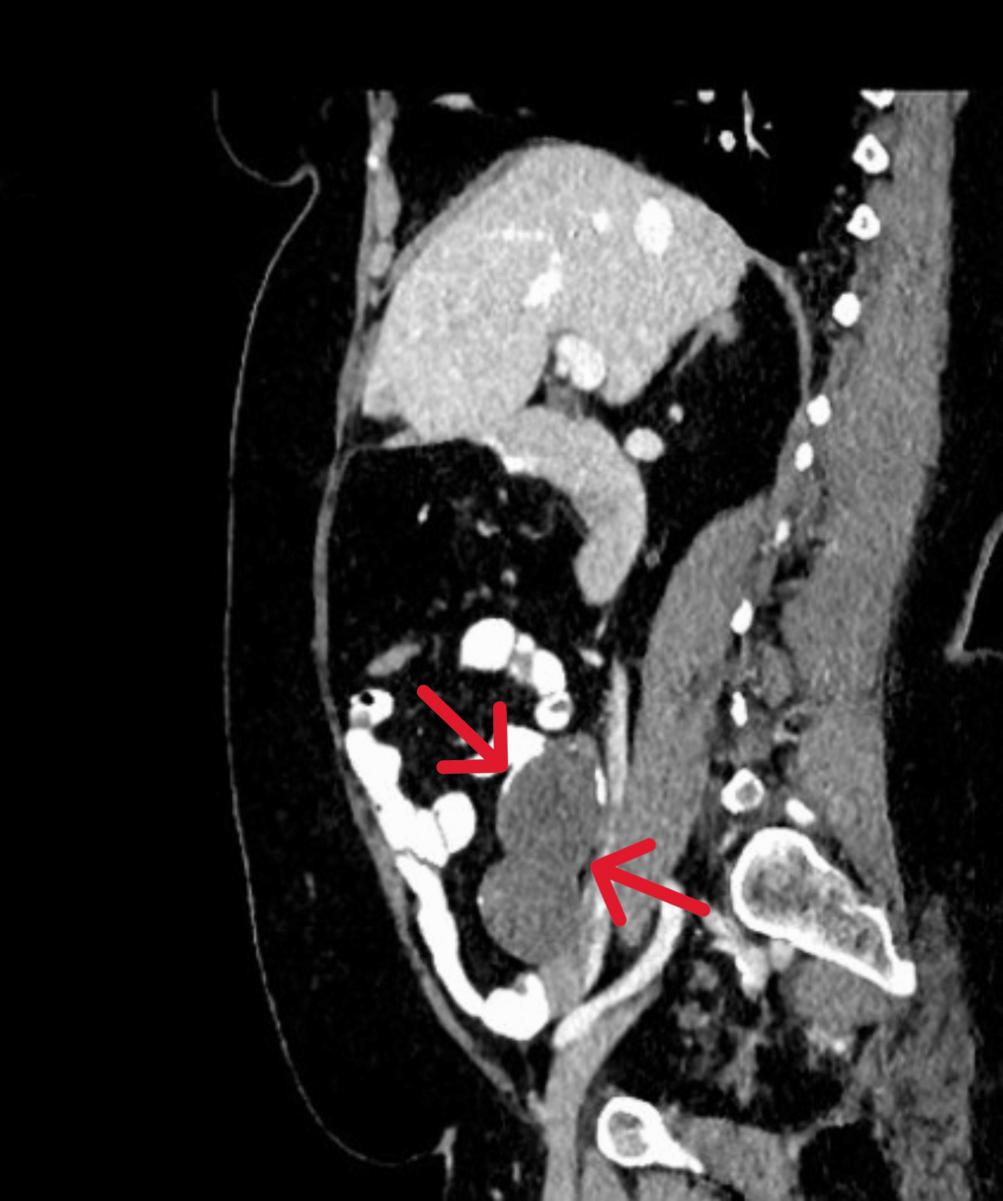
CT of the abdomen with contrast demonstrating hypodense appendiceal mucocele in sagittal view.

A 52-year-old female presented for an average risk-screening colonoscopy with no gastrointestinal symptoms and a benign medical history. During the procedure, a cecal bulge was observed originating from the appendiceal orifice (Figure 1). There was no visible associated inflammation or colitis, and the remainder of the colonoscopy was within normal limits. A subsequent CT scan of her abdomen revealed findings consistent with an appendiceal mucocele with no evidence of peri-appendiceal fat-stranding (Figures 2, 3). The patient was referred to general surgery which recommended appendectomy. Following appendectomy, surgical pathology reported that the mucosa was extensively replaced by acellular mucinous material forming pools within the lumen, lined by flattened, benign-appearing epithelium. There was no evidence of epithelial dysplasia or neoplasia. The muscularis propria was thinned but intact, and no acute inflammation or signs of appendicitis were observed. Pathology findings confirmed the suspicion of appendiceal mucocele. Surgical margins were negative and the patient fully recovered. The patient was recommended to continue routine colonoscopy screening.

## Discussion

Appendiceal mucoceles first entered the medical literature when described by Austrian pathologist Dr. Rokitansky in 1842 [5]. In modern times, approximately 3,500 cases of appendiceal mucocele are reported annually in the United States [4]. Appendiceal mucoceles are most often benign, but due to the inability to discern dysplasia with the naked eye, pathology is the only true way to know a mucocele’s malignant potential. On gross inspection, appendiceal mucoceles are dilated due to mucin accumulation within the lumen [6]. In this case, dilation from the buildup of mucin was so extreme that the cecum was visibly enlarged and bulging on colonoscopy.

Although the patient in this case was asymptomatic, mucoceles can present clinically similar to appendicitis with symptoms of right lower quadrant pain or generalized abdominal discomfort. Because of their similar presentation, appendiceal mucoceles are often preoperatively misdiagnosed as appendicitis [2]. Gao et al. (2022) retrospectively analyzed the charts of 3,701 patients admitted for appendicitis and found that nine cases were appendiceal mucoceles, with two of the nine being low-grade appendiceal neoplasms on histologic examination. All nine patients with mucoceles were successfully treated with either appendectomy, cecectomy, right hemicolectomy, or some combination of these [2]. The American Society of Colon and Rectal Surgeons 2019 guidelines recommend that all patients with low-grade mucinous neoplasms of the appendix with “negative margins and no evidence of perforation or peritoneal involvement” should be treated with appendectomy [7]. Furthermore, care should be taken to remove the mucocele without perforation so as not to disseminate the potential malignancy in the abdomen. In some scenarios, converting a laparoscopic appendectomy to open is necessary to safely remove the tumor without perforation [7]. Aside from potential malignancy, appendiceal mucoceles should also be removed to prevent rare complications seen in the literature such as rupture with acute abdomen, irreducible ventral hernia, appendiceal torsion, intussusception into the cecum, small bowel obstruction, and ureteral obstruction [8-13].

A brief review of the literature found a few cases of appendiceal mucocele discovered through colonoscopy. Several cases were of patients being investigated for recurrent abdominal pain [14-16]. One case similarly was asymptomatic and found on routine colonoscopy screening; however, the mucocele only involved up to the mid-appendix with no signs of cecal involvement [17]. Taj et al. (2013) had a very similar case to ours in terms of the discovery of a cecal bulge on colonoscopy but the patient was being investigated for chronic diffuse abdominal pain [15]. Our case is unique in that the mucocele, despite compressing the cecum and causing a visible mass effect, was entirely asymptomatic and found on routine colonoscopy screening.

## Conclusions

Appendiceal mucoceles are rare tumors that may present with symptoms similar to appendicitis such as right lower quadrant pain or may be asymptomatic and found incidentally. Due to the inability to differentiate benign mucoceles from malignancy based on imaging and the potential for complications, appendectomy with surgical pathology is recommended. No further intervention is recommended for patients with a simple appendiceal mucocele or low-grade appendiceal mucocele with negative surgical margins.
